# New methods to investigate the GnRH pulse generator

**DOI:** 10.1530/JME-23-0079

**Published:** 2024-01-11

**Authors:** Deyana Ivanova, Kevin T O’Byrne

**Affiliations:** 1 Department of Women and Children’s Health, Faculty of Life Science and Medicine, King’s College London, UK; 2 Division of Endocrinology, Diabetes, and Hypertension, Brigham and Women’s Hospital, Harvard Medical School, Boston, Massachusetts, USA

**Keywords:** hypothalamus and neuroendocrinology, neurotransmitters, female reproduction, male reproduction

## Abstract

The exact neural construct underlying the dynamic secretion of gonadotrophin-releasing hormone (GnRH) has only recently been identified despite the detection of multiunit electrical activity volleys associated with pulsatile luteinising hormone (LH) secretion four decades ago. Since the discovery of kisspeptin/neurokinin B/dynorphin neurons in the mammalian hypothalamus, there has been much research into the role of this neuronal network in controlling the oscillatory secretion of gonadotrophin hormones. In this review, we provide an update of the progressive application of cutting-edge techniques combined with mathematical modelling by the neuroendocrine community, which are transforming the functional investigation of the GnRH pulse generator. Understanding the nature and function of the GnRH pulse generator can greatly inform a wide range of clinical studies investigating infertility treatments.

## Introduction

Over 40 years ago, Ernst Knobil and colleagues detected the electrophysiological manifestation of the neural ensemble underlying the gonadotrophin-releasing hormone (GnRH) pulse generator in the arcuate nucleus of the hypothalamus (ARC) in rhesus monkeys (Knobil 1980, Wildt *et al.* 1981, O’Byrne & Knobil 1993). These early electrophysiological recordings consisted of abrupt increases in multiunit electrical activity (MUA) volleys invariably associated with pulsatile luteinising hormone (LH) secretion (O’Byrne & Knobil 1993); however, the phenotype of the neuronal networks forming the GnRH pulse generator remained elusive. Тhe late 20th century revealed that endogenous opioid peptide signalling is involved in mediating the steroid hormone negative feedback regulation of GnRH pulse generation in the ewe (Goodman *et al.* 1995). Although, other early reports conducted in humans concluded that endogenous opioid peptide signalling is somewhat involved in the regulation of LH secretion during the menstrual cycle, as naloxone administration had little effect on LH release during the early follicular phase but influenced the pattern of LH secretion during the high oestrogen and oestrogen–progesterone phases of the menstrual cycle (Quigley & Yen 1980).

Nevertheless, the role of endogenous opioid peptide in suppressing GnRH pulse generator frequency was more recently replicated in human patients where antagonism of endogenous opioid peptide signalling increased the frequency of LH pulses (Lippincott *et al.* 2019). In the 2000’s, kisspeptin (kiss1) was found to be the most potent stimulator of GnRH secretion in sheep (Goodman *et al.* 2007), monkeys (Shahab *et al.* 2005), and humans (Dhillo *et al.* 2005). Furthermore, investigations into patients with precocious puberty revealed the presence of activating mutations in the genes expressing kiss1 and the kiss1 receptor (Teles *et al.* 2008, Silveira *et al.* 2010) while inactivating mutations in the genes encoding the kiss1 receptor led to hypogonadotropic hypogonadism (De Roux *et al.* 2003, Seminara *et al.* 2003). Additionally, a high density of kiss1-expressing neurons was discovered in the ARC (Lehman *et al.* 2013), the site of MUA volley recordings, where most of these neurons are found to express steroid receptors crucial for steroid hormone feedback by oestrogen, progesterone and testosterone (Foradori *et al.* 2002, Smith *et al.* 2005, Franceschini *et al.* 2006). These observations combined provided compelling evidence that the interaction between kiss1 and GnRH neurons is essential for maintaining dynamic GnRH and LH release required for fertility in mammals. Crucially the application of multiple-label immunofluorescence studies in the ewe revealed that the majority of ARC kiss1 neurons co-express tachykinin neurokinin B (NKB) and the endogenous opioid peptide dynorphin (Goodman *et al.* 2007), which was consistent with other immunofluorescence observations made in the rat (Burke *et al.* 2006) and sheep (Foradori *et al.* 2006). Importantly, inactivating mutations in human genes encoding NKB and its receptor, neurokinin 3 (NK3R), result in defects in GnRH release and subsequent hypogonadism (Topaloglu *et al.* 2009), resembling the neuroendocrine profile of human and mouse mutations in the genes encoding the kiss1 receptor. Although, the mechanisms by which NKB signalling influences the function of the reproductive axis are more complex. Initially selective activation of NK3R in ovariectomised rats with oestradiol replacement suppressed LH secretion (Sandoval-Guzmán & Rance 2004). Contrastingly, subsequent reports showed a stimulatory effect on LH secretion following administration of selective NK3R agonist, senktide, in mice (Navarro *et al.* 2009), rats (Navarro *et al.* 2011), goats (Wakabayashi *et al.* 2010), sheep (Goodman *et al.* 2013) and monkeys (Ramaswamy *et al.* 2010, 2011). The observation that kiss1 infusion restored LH pulses in humans with inactivating mutations in the genes encoding NKB and its receptor (Young *et al.* 2013) supported data from prepubertal monkeys showing that there is an increase in LH secretion associated with NKB signalling, which is dependent upon kiss1 signalling (Ramaswamy *et al.* 2011). The physiological role of this mechanism involving NKB signalling is still under debate, as mice lacking functional NK3R are capable of pubertal development and fertility (Yang *et al.* 2012), possibly due to the recruitment of compensatory mechanisms. Moreover, a recent study showed that both mice and humans lacking NKB demonstrate impaired LH secretion, which can be augmented by opioid antagonism (Lippincott *et al.* 2019) suggesting that NKB and dynorphin have opposing roles in regulating GnRH pulse frequency. The authors concluded that NKB and dynorphin signalling is inessential for GnRH pulse generation, as LH pulses were preserved. Both stimulatory and inhibitory effects of NKB on the mammalian reproductive axis have been observed depending on the species studied and the circulating gonadal steroid milieu highlighting the significance of unravelling the underlying mechanisms in a range of species and different physiological states. Regardless there exists a high degree of colocalisation of these three peptides in the ARC of many species (Navarro *et al.* 2009, Wakabayashi *et al.* 2010, True *et al.* 2011, Hassaneen *et al.* 2016), including non-human primates (Ramaswamy *et al.* 2010), this neuronal population was abbreviated as ‘KNDy’ (kisspeptin/neurokinin B/dynorphin). Although, the extent of dynorphin expressing cells present in the human infundibular nucleus remains unclear, as immunohistochemical labelling shows that dynorphin-positive cell bodies are scarce in this region of young human males (Hrabovszky *et al.* 2012) whereas prodynorphin mRNA is observed within the infundibular nucleus of pre- and postmenopausal women (Rometo & Rance 2008). Nevertheless, the impressive collective efforts of the neuroendocrine community to interrogate the identity and function of the GnRH pulse generator has led to the formation of the ‘KNDy hypothesis’. In this model, KNDy neurons are proposed to constitute the GnRH pulse generator, forming a neuronal network that facilitates autocrine feedback on the KNDy cell body. The synchronous activity of the KNDy network is coordinated by the action of glutamate, NKB and dynorphin resulting in the pulsatile release of kiss1 onto the GnRH dendron (Voliotis *et al.* 2021). The progressive development and application of cutting-edge tools applied in combination, such as genetic mouse models, *in vitro* electrophysiology, optogenetic techniques and calcium imaging strategies, has led to substantial progress in our attempts to unravel the nature and function of the GnRH pulse generator.

## Electrophysiological investigation of the GnRH pulse generator

Electrophysiological investigation into the cellular and molecular mechanisms governing the function of the GnRH pulse generator has played a pivotal role in our understanding of what drives and maintains its oscillatory nature. In the last two decades, these studies have significantly helped lay the foundation for the formation of the ‘KNDy hypothesis’. The electrophysiological properties of ARC KNDy neurons in mice have been previously described to exhibit high input resistance, expression of H-currents (necessary for burst firing) and persistent sodium current (Kelly *et al.* 2013, Zhang *et al.* 2013, Piet *et al.* 2015*a*). On the most part, KNDy neurons have been shown to either not exhibit spontaneous firing or to fire at very low rates of below 0.1 Hz. The remainder of KNDy neurons have been shown to have irregular firing patterns as well as a wide variety of firing rates ranging from 0.5 Hz to up to 5 Hz, depending on the study (Gottsch *et al.* 2011, De Croft *et al.* 2012, Alreja 2013, Frazão *et al.* 2013, Ruka *et al.* 2013, Mendonça *et al.* 2018, Wang *et al.* 2018, 2019).

The electrical properties of ARC KNDy neurons have been further characterised in brain slices from male mice using targeted whole-cell patch-clamp experiments (Mendonça *et al.* 2018). KNDy neurons from intact male mice exhibit an intrinsic irregularity of firing. The firing properties of KNDy neurons are influenced by fast inactivating potassium currents. Pharmacological blockade and kinetics combined with single-cell RT-PCR demonstrated that the A-type current is mediated mainly by Kv4.2 channels in these cells. The A-type current is an important determinant of KNDy neuronal firing dynamics and increases the firing irregularity of these neurons in response to depolarising current injections. The A-type current interacts with the persistent sodium current in KNDy neurons creating inter-spike voltage fluctuations, which affects the timing of spikes and diversifies the firing pattern. Importantly, variation in the density of Kv4 conductance is linked to the heterogeneity of spiking irregularity of KNDy neurons. This implies a natural fine-tuning mechanism due to adjustment of Kv4 kinetics likely determining the connectivity and role of each neuron in the KNDy network.

Kiss1 itself appears to have no effect on KNDy neuron electrical activity in brain slices from mice (De Croft *et al.* 2013) and ARC KNDy neurons do not express the kiss1 receptor (Herbison *et al.* 2010, Higo *et al.* 2017), indicating that kiss1 does not act directly upon KNDy neurons to regulate pulsatile reproductive hormone secretion in mice. The synaptic mechanisms involved in mediating the synchronous activity of ARC KNDy neurons were characterised using whole-cell electrophysiology, molecular pharmacology and single cell RT-PCR in brain slices from mice (Qiu *et al.* 2016). High-frequency optogenetic activation of ARC KNDy neurons induced a depolarisation, which was defined as the slow excitatory post-synaptic potential (EPSP). The slow EPSP was demonstrated to be mediated by *Tacr3* signalling via pharmacological and optogenetic manipulation of the receptor on KNDy neurons. Moreover, the slow EPSP is dependent on direct synaptic input from neighbouring ARC kiss1 neurons. Labelling with ChR2 revealed that KNDy neuronal fibres crossed over to the contralateral ARC and optic stimulation evoked fast-excitatory postsynaptic currents as well as the slow EPSP in these contralateral neurons. Additionally, the inhibition of glutamatergic and NKB-mediated excitatory input was shown to be mediated by pre-synaptic dynorphin signalling. These observations are largely supported by previous studies where intra-ARC administration of a κ-opioid receptor agonist has been shown to inhibit pulsatile LH secretion in rats (Grachev *et al.* 2012). In contrast, a recent report showed that conditional knockout of the κ-opioid receptor selectively in kiss1 neurons of male and female mice had no effect on puberty, LH pulse parameters, LH surges, follicle-stimulating hormone (FSH) levels, oestrous cycles or fertility (Coutinho *et al.* 2022). These data suggest that the KNDy network may not be the only neural mechanism involved in controlling GnRH and LH pulses in mice and humans. The suppressive action of dynorphin signalling on the electrical activity of KNDy neurons has been demonstrated *in vitro* (De Croft *et al.* 2013, Ruka *et al.* 2013, 2016). Furthermore, MUA volley recordings from the ARC show that neuronal activity is modulated by NKB and dynorphin, but remains unresponsive to kiss1 and GnRH, in accordance with the KNDy hypothesis (Hiruma & Kimura 1995, Ördög *et al.* 1997, Liu *et al.* 2009, Ohkura *et al.* 2009, Wakabayashi *et al.* 2013). On the other hand, norBNI, a κ-opioid receptor antagonist, failed to affect spontaneous electrical activity of KNDy neurons in mice, despite the fact it was able to block the inhibitory actions of dynorphin in this preparation (De Croft *et al.* 2013, Ruka *et al.* 2013). Moreover, norBNI also appeared to have no effect on LH pulse frequency when administered into the ARC (Grachev *et al.* 2012) or into the third ventricle (Mostari *et al.* 2013), but this could be due to a species difference, as these studies were performed in rats. Nevertheless, there is compelling evidence that the cellular interactions generating episodes of synchronised KNDy neuron activity, which are relayed to GnRH neurons through kiss1, involve the excitatory effect of NKB as well as the inhibitory effect of dynorphin.

More recently, Kelly and colleagues employed optogenetics with whole-cell recordings and calcium imaging in brain slices to further explore the cellular mechanisms underlying the synchronous activity of KNDy neurons (Qiu *et al.* 2021). Since the majority of KNDy neurons express stromal interaction molecule 1 (STIM1), the interaction between *Tacr3* and TRPC5 channels was found to be modulated by STIM1 in these neurons. The role of STIM1 in KNDy neurons is to replenish the endoplasmic reticulum upon depletion of calcium. From the previous study, we know that *Tacr3* is greatly involved in generating a robust inward current, leading to the depolarisation of KNDy neurons and increasing their excitability. KNDy neurons also contain TRPC5 channels, which further contribute to the generation of the inward current underlying the slow EPSP. The application of BAPTA, a calcium chelator, was found to inhibit the slow EPSP proving that TRPC5 channels are critical to the intrinsic bistability that generates the persistent firing activity of KNDy neurons. STIM1 deletion from KNDy neurons elevates calcium influx into the cell while enhancing the amplitude and duration of the slow EPSP. In the presence of oestrogen, STIM1 is downregulated; thus, in a reproductively active female where STIM1 expression is low, TRPC5 channels are converted from store-operated channels to receptor-operated channels. In this case, *Tacr3* facilitates TRPC5 channel opening, leading to an increase in KNDy neuronal excitability.

**Figure 1:**
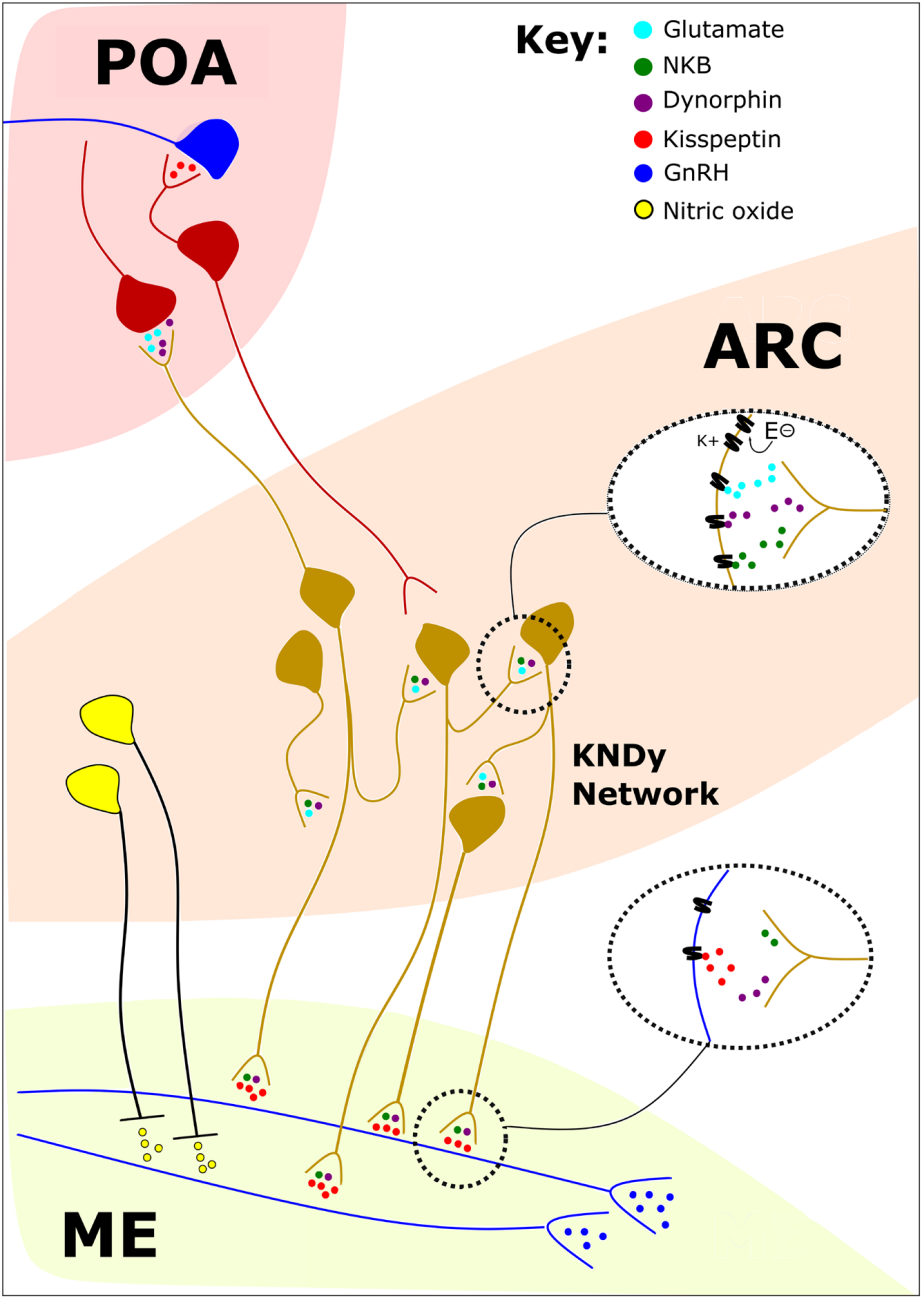
The GnRH pulse generator. The circuit dynamics regulating the episodic activity of the KNDy neuronal network located in the arcuate nucleus of the hypothalamus (ARC) involve the action of glutamate (cyan), neurokinin B (NKB) (green), and dynorphin (purple) signalling onto glutamatergic, neurokinin 3 and kappa opioid receptors, respectively, within the network. Voltage-gated potassium channels and oestrogen receptors on KNDy neurons further modulate the activity of the network. Only kiss1 release from KNDy fibres activates the distal gonadotrophin-releasing hormone (GnRH) dendrites despite the potential release of all neuropeptides from these neurons and stimulate the release of GnRH in the median eminence (ME). KNDy neurons regulate nitric oxide expressing neurons in the ARC providing a global reset of the signalling pathway components in GnRH neurons required for the next episode of kiss1 stimulation. There is functional synaptic contact between KNDy and kiss1 neurons in the preoptic area (POA). KNDy neuron activity amplifies the LH surge via glutamate and dynorphin release onto POA kiss1 neurons. Kiss1 neurons in the POA release kiss1 onto GnRH soma in this region to modulate the activity of GnRH neurons. GnRH (blue), KNDy (gold), Nitric oxide (yellow), Kiss1 (red).

The KNDy model provides an on-signal for GnRH neuron activation, triggering GnRH pulses with the release of kiss1; however, it is thought that this model lacks an off-signal for GnRH neurons. Locally applied kiss1 on GnRH nerve terminals has been previously shown to evoke a long-lasting increase in intracellular calcium levels (Iremonger *et al.* 2017). KNDy neurons trigger the pulsatile release of GnRH; however, the inhibitory action of dynorphin on the oscillatory dynamics of the KNDy network is not necessarily obligatory for the termination of GnRH pulses (Grachev *et al.* 2012, Mostari *et al.* 2013). Moreover, it has become increasingly evident that dynorphin expression is mainly absent from kiss1-NKB expressing neurons in humans (Hrabovszky *et al.* 2012, Anderson & Millar 2022). Although, there is evidence that kiss1 is released in a pulsatile manner in the vicinity of the median eminence before most GnRH pulses in pubertal monkeys (Keen *et al.* 2008). Calcium imaging combined with electrophysiology in slice has been employed to uncover the cellular mechanisms that allow intracellular calcium levels in GnRH neurons to return to baseline following repetitive stimulation with kiss1 (Constantin *et al.* 2021). There is a close anatomical relationship between kiss1 fibres and nitric oxide expressing neurons in the ARC of non-human primates, sheep and mice (Hanchate *et al.* 2012, Bedenbaugh *et al.* 2018*a*,*b*) while nitric oxide expressing neurons are also found in the infundibular nucleus of humans (Egberongbe *et al.* 1994). The ongoing activity of TRPC channels maintains the calcium response in GnRH neurons from kiss1 stimulation. Nitric oxide exposure was found to facilitate the subsequent response to kiss1 while deactivating TRPC channels and restoring their availability (Constantin *et al.* 2021). In this proposed model, nitric oxide expressing neurons located in the ARC are activated by KNDy neurons and the highly diffusible nitric oxide is released in the vicinity of GnRH neurons providing a global reset of the signalling pathway components in GnRH neurons required for the next episode of kiss1 stimulation (Fig. 1) (Constantin *et al.* 2021).

Importantly, the KNDy network is steroid sensitive and utilises glutamatergic transmission for intra-network communication (Qiu *et al.* 2016, 2018). A study using the CRISPR-Cas9 approach to knock down oestrogen receptor α (ERα) selectively in ARC KNDy neurons combined with electrophysiological analysis found an increase in glutamatergic transmission to these neurons while disrupting cycles (Wang *et al.* 2019), like previous observations made in kiss1 cell-specific ESR1 knockout (KERKO) mice (Wang *et al.* 2018). Since oestradiol feedback is likely partly relayed to GnRH neurons via ERα-expressing ARC KNDy neurons, a follow-up study demonstrated that the oestradiol-sensitive intrinsic properties and potassium conductance regulate the membrane potential response to fast synaptic input in ARC KNDy neurons (DeFazio *et al.* 2019). Oestradiol has been shown to elevate the depolarisation response to GABA_A_ receptor activation in KNDy neurons (Defazio *et al.* 2014). This led to the idea that voltage-gated conductances intrinsic to KNDy neurons are oestradiol-sensitive, which contributes to enhancing the membrane potential response to synaptic inputs. Whole-cell recordings of ARC KNDy neurons in the presence and absence of oestradiol in mice revealed that oestradiol reduced voltage-gated potassium currents and action potential latency (DeFazio *et al.* 2019). Moreover, the use of dynamic clamp to simulate GABA and AMPA conductance showed that GABA and AMPA induced postsynaptic potentials were smaller in the absence of oestradiol and blocking transient potassium currents eliminated this effect, supporting previous work demonstrating that the transient potassium current in KNDy neurons contribute to firing irregularity (Mendonça *et al.* 2018). These studies indicate that voltage-gated potassium currents in KNDy neurons are modulated by oestradiol and interact with intrinsic and extrinsic properties to modify the firing output of these neurons.

In summary, recent electrophysiological studies have shed light onto the electrical properties of KNDy neurons and the cellular mechanisms involved in modulating the oscillatory nature of the KNDy network. KNDy neurons exhibit an intrinsic irregularity of firing and fast inactivating potassium currents influence the electrical properties of these neurons. Voltage-gated potassium channel activity is important for determining the connectivity and role of each neuron in the KNDy network (Fig. 1). It is well known that kiss1 does not act directly upon KNDy neurons to regulate pulsatile reproductive hormone secretion. Investigation into the complex synaptic mechanisms engaged to regulate the episodic activity of KNDy neurons confirmed that excitatory inputs within the KNDy network are mediated by glutamate and NKB signalling, and inhibition of these excitatory inputs is mediated by pre-synaptic dynorphin signalling. Moreover, the excitability of KNDy neurons was found to be regulated by STIM1, which is another crucial component involved in modulating the synchronous firing of KNDy neurons. The output signal produced from KNDy network activity is the release of kiss1, and this is proposed to provide an on-signal to trigger pulsatile GnRH secretion, but it is thought that this model lacks an off-signal. Nitric oxide expressing neurons in the ARC were proposed to provide a global reset of the signalling pathway components in GnRH neurons required for the next episode of kiss1 stimulation. The KNDy network is steroid sensitive and knockdown of ERα selectively in ARC KNDy neurons increased glutamatergic transmission to these neurons, indicating that oestradiol regulates intra-network communication. Furthermore, oestradiol modulates voltage-gated potassium currents in KNDy neurons, which modifies the firing output of each neuron in the network.

## Application of optogenetic and chemogenetic strategies to study the GnRH pulse generator

In 2015, Han *et al.* published the first study employing optogenetics to explore whether the synchronised activation of ARC kiss1 neurons leads to pulsatile LH secretion (Han *et al.* 2015). It was discovered that stimulation frequencies of 10 Hz and above over a period of 2–5 min can induce an LH pulses in ovariectomised female and intact isoflurane-anesthetised mice. The application of isoflurane in this study blocked endogenous LH pulses; thus, pulsatile LH secretion was driven by the high-frequency short burst photostimulation of ARC kiss1 neurons. Lower frequency stimulation of these neurons at 2 Hz over 5 min failed to induce LH pulses, which led to the idea that the sole release of neurotransmitters from these neurons, specifically glutamate, are insufficient in modifying pulsatile LH secretion (Liu *et al.* 2011, Han *et al.* 2015). Clarkson and colleagues then went on to provide further evidence that ARC KNDy neurons form the core of the GnRH pulse generator, as an extrinsic driver of GnRH pulses, using optogenetics in awake mice (Clarkson *et al.* 2017). Photostimulation of KNDy neurons at 10 Hz for 1 min was able to generate LH pulses and this was repeatable at 30-min intervals in intact mice. Moreover, archaerhodopsin-mediated silencing of KNDy neuronal activity resulted in reduced LH pulse amplitude and frequency while activation of halorhodopsin with a constant green-light illumination of these neurons was able to reset pulsatile LH secretion.

In 2019, a study combining optogenetics and mathematical modelling to investigate the circuit mechanisms enabling the pulsatile dynamics of KNDy neurons highlighted that the KNDy network undergoes qualitative changes in its oscillatory behaviour as the basal activity of the population is modulated (Voliotis *et al.* 2019). The sustained application of blue light between 1 and 15 Hz induced regular LH pulses in oestrous mice, which normally display minimal intrinsic pulse generator activity, while anything above or below this optic light frequency range failed to evoke LH pulses. Importantly, optic stimulation at 1 Hz was able to initiate sustained pulsatile LH secretion and the frequency of oscillation was increased with the application of 5 Hz photostimulation, indicating the critical involvement of neurotransmitter action in triggering synchronous activity within the KNDy network, as low-frequency optic stimulations typically evoke solely neurotransmitter release (Arrigoni & Saper 2014). The model then predicted a decrease in the frequency of oscillation with higher activation frequencies and this was observed experimentally where optic stimulation at 15 Hz decreased the frequency of LH pulses. This characteristic tipping point behaviour of the system emerges due to nonlinear positive and negative feedback interactions modulated by NKB and dynorphin signalling within the network, which was confirmed with a combination of pharmacological manipulation and optogenetics. In other words, as the basal activity level is increased the network transitions from a silent into a pulsatile mode, while higher levels of basal activity suppress pulses and reinstate the quiescent state. Importantly, these sudden transitions are a fundamental characteristic of the mechanisms underlying pulse generation per se.

A different study examining the temporal frequency constraints within which the GnRH pulse generator can drive LH pulses found that optic stimulation of KNDy neurons at 10 Hz for 1 min every 45 min generated an LH pulse in response to each pulse of optic stimulation. With this optic stimulation protocol the pattern of pulsatile LH secretion was similar to intact mice while a faster rate of optic stimulation, every 9 min at the same frequency, produced an LH pulse profile like that of gonadectomised mice (Han *et al.* 2020). On the other hand, optic stimulation of KNDy neurons at 10 Hz every 3 min resulted in a nonpatterned LH pulse profile with gradually declining LH levels. The presence of gonadal steroids is known to alter the function of many neuropeptides and neurotransmitters (Kordon *et al.* 1994), particularly those engaged by KNDy neurons to regulate network dynamics; thus, the authors tested whether a stimulatory input to these neurons would result in a different effect on LH pulses in intact and gonadectomised mice. Photostimulation of KNDy neurons with 10 Hz every 3 min for 1 h led to gonadotroph desensitisation and the collapse of pulsatile LH release in gonadectomised mice. The same optic stimulation protocol was applied in intact mice, which exhibit a slower endogenous pulse frequency rate, and GnRH pulse generator frequency was maintained within its operational range.

Recent mathematical modelling has shown that excitability within the KNDy network is regulated by the circulating gonadal steroid milieu across the ovarian cycle (Voliotis *et al.* 2021). Low-frequency optic stimulation of KNDy neurons accelerates pulsatile LH secretion during oestrous, whereas the opposite occurs during dioestrus potentially due to changes in glutamate signalling within the network. Gonadal steroids play a role in shifting the lower and upper bifurcation points, which define the system’s range of pulsatile behaviour. Since KNDy neurons are glutamatergic and synapse onto each other (Qiu *et al.* 2016), network excitability is likely enabled by the action of glutamate within the network. Using a dual optic stimulation and drug delivery system, the authors showed that in low oestrogen conditions, such as during diestrus, blocking glutamate signalling in the ARC inhibited endogenous LH pulses, which were rescued with 5 Hz optic activation of KNDy neurons. Contrastingly, under high oestrogen conditions, blocking glutamatergic signalling in the ARC prevents the initiation of LH pulses by optic stimulation. This suggests that circulating gonadal steroids influence glutamate activity, which mediates inter-neuronal communication vital for the synchronous activity of the KNDy network.

Interestingly, sustained low-frequency activation of ARC KNDy neurons also induces a surge-like increase in LH secretion in prooestrous and oestrogen-replaced ovariectomised mice (Lin *et al.* 2021). Thus, KNDy neurons may release glutamate onto AVPV kiss1 neurons via the functional synaptic contact between the two neuronal populations (Qiu *et al.* 2016) subsequently exerting a facilitatory role in the production of the LH surge. Although, previously, it has been shown that ablation of KNDy neurons in the ARC with saporin-conjugated neurokinin B receptor agonist injections increased the steroid-induced LH surges, which was accompanied by an increase of kiss1 content in the AVPV in oestradiol-treated ovariectomised rats (Helena *et al.* 2015). It was proposed that the lack of dynorphin input from KNDy neurons to the AVPV was responsible for these effects. Therefore, dynorphin antagonist was administered into the AVPV and that appeared to significantly increase the LH surge, like KNDy neuron ablation, while intra-AVPV administration of dynorphin in KNDy-ablated rats seemed to restore LH surge levels. It is common for peptide action in the CNS to be involved in the modulation of fast synaptic activity and it is often the case that neuropeptides regulate neurotransmitter signalling at pre- or postsynaptic sites (van den Pol 2012). Since both glutamate and dynorphin signalling exists between the ARC KNDy and AVPV kiss1 neuronal populations, it is possible that dynorphin is acting to modulate glutamatergic transmission between these two neuronal sites, in turn, regulating the production of the LH surge (Fig. 1). Intriguingly, the use of expansion microscopy has revealed that the density of synaptic inputs at the distal GnRH dendrite is larger than that of the proximal dendrite; thus, it is the most densely innervated dendritic compartment of the GnRH neuron (Wang *et al.* 2020). Moreover, selective chemogenetic inhibition of the distal GnRH dendrite reduces pulsatile LH secretion and suppresses the LH surge. This indicates that synaptic integration at the GnRH distal dendron is also required for the generation of the LH surge.

Optogenetic investigation of the GnRH pulse generator has greatly advanced our understanding of the functional neuronal mechanisms regulating the oscillatory behaviour of the KNDy network in awake freely behaving mice. These pioneering studies provide direct evidence that KNDy neurons constitute the GnRH pulse generator and defined the operational range of these neurons as well as the pulsatile dynamics of the KNDy network. The development of mathematical models to help resolve the mystery of what initiates GnRH pulse generation predicted that modulation of the basal activity of the population induces changes in the oscillatory behaviour of the KNDy network. This was shown experimentally using different frequencies of sustained optogenetic stimulation to drive the endogenous activity of KNDy neurons in oestrous mice. Additionally, NKB was shown to provide positive feedback and dynorphin slow negative feedback to generate robust oscillation of neural activity in KNDy neurons, which is supported by electrophysiological data. The application of different optogenetic stimulation protocols to KNDy neurons contributed to describing the system further by revealing the temporal constraints within which the pulse generator operates to drive efficient LH pulsatility. The mathematical model of the system was later expanded to include the effect of fluctuating levels of gonadal steroids across the ovarian cycle on the oscillatory behaviour of the KNDy network. The model combined with an optogenetic approach confirmed the idea that the KNDy network is steroid sensitive, as circulating gonadal steroids were found to influence glutamate activity, which mediates inter-neuronal communication vital for the synchronous activity of the KNDy network. Interestingly, KNDy neuron activity was also shown to amplify the LH surge likely occurring via existing functional synaptic contact between KNDy and AVPV kiss1 neurons. Additionally, the application of chemogenetic strategies revealed that synaptic integration at the GnRH distal dendron is required for generation of the LH surge and regulation of LH pulses.

## Investigating the function of the GnRH pulse generator using calcium imaging

The progressive development of calcium imaging techniques has recently become essential for dissecting the functional neural networks controlling episodic reproductive hormone secretion. Following the discovery of ARC KNDy neurons as a crucial component of the GnRH pulse generator, the application of calcium imaging *in vitro* and *in vivo*, at the neuronal population (fibre photometry) and single-cell (gradient-index (GRIN) lens mini-endoscopy) level, has revealed a pattern of periodic synchronisation invariably associated with pulsatile LH secretion.

### In vitro

Measuring calcium signals in specific cell types *in vitro* with a high degree of temporal and spatial resolution provides a powerful tool for uncovering the role of calcium in these cells. Intracellular calcium signalling is also an approximate indicator of the electrical activity of these cells. Although detecting calcium activity within cells is currently the best available proxy for monitoring neuronal firing, it will not always perfectly reflect the electrical activity of neurons, which is especially true for single spikes. Earlier studies applying calcium imaging *in vitro* to study GnRH and kiss1 neurons used calcium indicators such as pericam (Jasoni *et al.* 2007), GCaMP3 (Piet *et al.* 2015*b*) and GCaMP6 (Clarkson *et al.* 2017, Iremonger *et al.* 2017). The use of GCaMPs can be combined with Cre-LoxP recombination approaches, which allows for the conditional expression of these indicators in distinct genetically defined neuronal populations in specific brain regions (Partridge 2015). Previous reports show a reasonable temporal relationship between GCaMP-monitored calcium changes and neuronal electrical activity in burst firing neuroendocrine neurons (Piet *et al.* 2015*b*, Iremonger *et al.* 2017). The selective expression of GCaMP6 in GnRH neurons allowed for the recording of intracellular calcium activity in the GnRH distal dendrons and nerve terminals (Iremonger *et al.* 2017). This strategy permitted access to these remote locations where it is not practically feasible to make electrical recordings. These types of experiments requisite the use of confocal imaging and can be time-consuming but provide invaluable insight into the temporal patterns of action potential- and neurotransmitter-activated calcium release at these sites.

Genetically encoded calcium indicators combined with single-cell imaging in a neonatal organotypic slice culture model has been used to examine endogenous calcium oscillations in KNDy neurons as well as the regulatory components contributing to calcium oscillation in these neurons’ *ex vivo* in mice (Kim *et al.* 2020). This type of *ex vivo* model preserves functional features of the original tissue, although *ex vivo* incubation can also introduce alterations in neuronal characteristics. KNDy neurons were seen to exhibit self-sustained synchronised calcium oscillations in the neonatal stage where voltage-gated sodium and potassium channels contributed to the generation of synchronous calcium oscillations in these neurons, indicating the involvement of action potentials. Using a chemogenetic approach the authors showed that signalling through NK3R and κ-opioid receptor did not seem to influence calcium oscillations in KNDy neurons, although the expression levels of these receptors in neonatal slices was shown to be low possibly since *Tac2* and *Tacr3* expression increases with postnatal maturation (Gill *et al.* 2012, Navarro *et al.* 2012). Despite the lack of NKB and dynorphin regulation within the KNDy network in neonatal slices synchronised calcium oscillations were still present, indicating that KNDy neurons may generate autonomous oscillatory behaviour during the early stages of development. Since gonadectomy increases the frequency of calcium oscillations in KNDy neurons of male mice over time (Han *et al.* 2019), the activity of the KNDy network likely reshapes alongside sexual development and the presence of the steroid milieu. Neural signalling via the NMDA and GABA_A_ receptor was found to be essential for maintaining the calcium oscillations in KNDy neurons (Kim *et al.* 2020).

Synchronisation of ARC KNDy neurons occurs in a kiss1-independent manner, but pulsatile LH secretion evoked by the oscillatory activity of the KNDy network depends on appropriate kiss1 output onto GnRH dendrons in the median eminence (Qiu *et al.* 2016, Voliotis *et al.* 2019, Liu *et al.* 2021). However, the nature of kiss1 signalling at the GnRH dendrons was unclear. A recent report using confocal analysis and expansion microscopy has revealed the nature of these appositions to be non-synaptic and instead they signal via volume transmission rather than conventional synapses to activate GnRH nerve terminals (Liu *et al.* 2021). Contrastingly, kiss1 neurons form genuine synapses at the GnRH soma and proximal dendrites (Yip *et al.* 2015, Qiu *et al.* 2016, Yeo *et al.* 2019). Expansion microscopy employs isotropic swelling of the tissue specimen, providing spatial resolution reliable for imaging synapses in the brain (Wassie *et al.* 2018). Previous electron microscopy studies performed in the rat median eminence support these findings, as they showed closely apposed kiss1 fibres and GnRH nerve terminals do not form synapses (Uenoyama *et al.* 2011). Thus, synchronous activation of GnRH dendrons is likely achieved by short-distance volume transmission arising from multiple KNDy neuron axons (Fig. 1). Further electrophysiology and GCaMP imaging (fibre photometry) showed that only kiss1 release from KNDy fibres activates the distal GnRH dendrites despite the potential release of all neuropeptides from these neurons (Fig. 1). Multi-barrelled pipettes were used to apply short puffs of candidate neuropeptides or neurotransmitters to GnRH dendrons in acute horizontal brain slices in which calcium activity can be measured simultaneously using confocal imaging. Short puffs of kiss1 induced a rise in calcium fluorescence in GnRH dendrons; however, NKB and dynorphin puffs of the same duration had no effect on calcium activity in the dendrons in male and female mice. Puffs of glutamate, NMDA or AMPA, were also found to have no effect on calcium fluorescence in the distal GnRH dendrons. Earlier findings demonstrate that puffs of glutamate did evoke responses in the GnRH dendrons (Herde *et al.* 2013), albeit this is possibly due to a falling gradient of glutamate receptor expression along the GnRH projection, as puffs of glutamate along the GnRH projections greater than 350 µm from the cell body were shown to be ineffective.

Electron microscopic imaging has revealed the presence of symmetric, stereotypical GABAergic, synapses on the GnRH distal dendron and the GnRH dendron is modulated by GABAergic transmission (Moore *et al.* 2018). The latest study using calcium imaging *in vitro* explored the functional GABAergic regulation of the GnRH distal dendrons. Using the same approach, Herbison and colleagues applied puffs of GABA on the GnRH dendrons and measured their calcium response using confocal imaging of acute brain slices (Liu *et al.* 2022). The GABA_B_ receptor was found to be the dominant subtype evoking a monophasic sustained suppression of calcium activity in the GnRH distal dendron in male and female mice, particularly in the prooestrous and oestrous stage of the cycle. Similarly, Kelly and colleagues had shown previously that GABA_B_ receptor activation at the GnRH soma exerts a hyperpolarisation effect mediated by G protein-gated inwardly rectifying potassium (GIRK) channels (Zhang *et al.* 2009). Functional GABA_A_ receptor expression was limited with little efficacy in males, although a stimulatory effect was observed in prooestrous females with 50% of GnRH dendrons responding with rapid increases in intracellular calcium. Activation of the GABA_A_ receptor on the GnRH dendron evoked a biphasic acute elevation in intracellular calcium levels followed by a suppression. Dendrons did not display a GABA_A_ response without a subsequent GABA_B_-mediated suppression, indicating GABA_A_ are colocalised with GABA_B_ receptors, whereas this was not the case for GABA_B_ receptors which often appeared to be the sole functional GABA receptor expressed by the dendron. Application of baclofen, a GABA_B_ receptor agonist, was also shown to suppress kiss1 evoked increases in intracellular calcium levels which are known to be dependent on voltage-gated calcium channels, indicating that the dominant GABA_B_ receptor may act to counterbalance the excitatory drive provided by KNDy neurons. This idea is consistent with existing evidence of increased GnRH pulse frequency in mice with a global knockout of the GABA_B_ receptor (Catalano *et al.* 2010, Di Giorgio *et al.* 2019). Interestingly, at the GnRH soma, the hyperpolarisation effects exerted by GABA_B_ receptor activation were abolished by kiss1 administration whereas co-application of baclofen after kiss1 administration did not completely reverse the kiss1-induced depolarisation of GnRH cell bodies (Zhang *et al.* 2009). Nevertheless, GABAergic modulation of the distal GnRH dendron may be operating as a bidirectional input filter whereby phasic GABA activity promotes, and tonic GABA activity inhibits, GnRH release.

In summary, calcium imaging of KNDy neurons revealed these neurons exhibit self-sustained synchronised calcium oscillations in a neonatal organotypic slice culture model. Signalling through the NMDA and GABA_A_ receptors was found to be essential for maintaining calcium oscillations in KNDy neurons at this stage. The release of kiss1 from KNDy fibres is necessary to activate the distal GnRH dendrons. This occurs via short-distance volume transmission arising from multiple KNDy neuron axons at the GnRH dendron. In addition, GABAergic signalling also contributes to modulating the distal GnRH dendron operating as a bidirectional input filter whereby phasic GABA activity promotes, and tonic GABA activity inhibits, GnRH release.

### In vivo

With substantial technical advancement in calcium imaging strategies, we are now able to monitor fluorescent signals emanating from specific GCaMP-targeted neuronal populations with superb temporal dynamics in conscious-freely behaving mice. This approach enabled intracellular calcium activity of KNDy neurons to be measured simultaneously as a population in real time. Brief synchronised episodes of calcium activity occurring every 5–10 min were observed in these neurons and preceded each LH pulse in gonadectomised male mice (Clarkson *et al.* 2017). The application of GCaMP fibre photometry *in vivo* has also made it possible to perform long-term recordings of the activity of ARC KNDy neurons in the presence and absence of circulating gonadal steroids in mice (Han *et al.* 2019, Goto *et al.* 2023). A recent study measured calcium signals in KNDy neurons under a scheduled light emission mode for 24 h in mice connected to the fibre photometry system (Han *et al.* 2019). Synchronised episodes of calcium activity were seen to be perfectly correlated with LH pulses in intact and gonadectomised mice, reinforcing the role of the KNDy network as the GnRH pulse generator. The inter-synchronisation episode interval in the absence of gonadal steroids was drastically reduced compared to intact mice. Although, a wide range of inter-synchronisation episode intervals were observed in both intact and gonadectomised mice, indicating an unpredictable stochastic pattern of GnRH pulse generator activity, which is supported by existing long-duration studies examining LH pulses in male humans and mice (Coquelin & Desjardins 1982, Spratt *et al.* 1988). Intriguingly, synchronised episodes recorded from male gonadectomised mice often occurred in doublets, triplets and quadruplets unlike intact mice that exhibit only singlet episodes. According to the current KNDy hypothesis, dynorphin signalling within the KNDy network acts to terminate the synchronised episodes; thus, multi-episode clusters occurring in the absence of gonadal steroids may be due to ineffective dynorphin signalling within the network. The application of continuous light emission mode photometry providing high 10 Hz temporal resolution data revealed the dynamics of a synchronised episode were altered whereby in gonadectomised mice the speed of onset and magnitude of episodes were elevated compared to intact mice. Long-term gonadectomised mice appeared to display synchronised episodes with six-fold higher levels along with multi-episode synchronisations compared to intact as well as short-term gonadectomised mice (Han *et al.* 2019). These changes in amplitude of the synchronised episodes as time progresses following gonadectomy may reflect elevating NKB synthesis and available releasable pools as well as elevated NK3R expression in KNDy neurons. Nevertheless, these temporal changes suggest independent mechanisms are possibly engaged to control different aspects of GnRH pulse generator function.

Following on from these observations, a study published in 2022 aimed to define the oestrogen negative feedback pathway modulating the activity of the KNDy network using GCaMP6 photometry combined with the CRISPR-Cas9 gene editing technology (McQuillan *et al.* 2022). Initially the authors observed that kiss1 cell specific ESR1 knockout (KERKO) intact female mice exhibit frequent clusters of synchronised episodes, which differed from intact control mice but showed high similarity to the synchronised episodes observed in ovariectomised control mice. CRISPR-Cas9 gene editing was then applied to selectively delete ESR1 from ARC KNDy neurons, as the use of KERKO mice comes with limitations, including the fact that ESR1 is deleted from kiss1-expressing cells in the body and normal pathways of oestradiol action are possibly altered during development in mice with an ESR1 deletion. Selectively deleting ESR1 from ARC KNDy neurons resulted in GnRH pulse generator activity equivalent to that of gonadectomised control mice. The LH pulse profiles of these mice exhibited high-frequency low-amplitude LH pulses possibly due to reduced pituitary responsiveness. In mice with the CRISPR-Cas9 deletion of ESR1 from KNDy neurons, only the pituitary remains under oestradiol negative feedback. In the absence of the ovary, the pituitary can faithfully follow a 20-min interval GnRH stimulation regimen. This interval of GnRH stimulation results in accelerated low-amplitude pulsatile LH secretion. Despite the advantages of using the CRISPR-Cas9 technology over standard recombinase mediated genetic approaches there are limitations (Peng *et al.* 2016). It is not possible to achieve a complete knockout of ESR1 from the target cells and each animal will exhibit a different degree of gene deletion due to the random nature of DNA repair mechanisms engaged following endonuclease cleavage. Thus, a variable degree of ESR1 deletion was achieved in this study and the results indicate that only 20–30% of ESR1-expressing KNDy neurons are sufficient to mediate the negative-feedback effects of oestrogen, indicating a high degree of redundancy within the KNDy neuronal network (McQuillan *et al.* 2022).

The mechanisms implemented in the origin of episodic activity of central pattern generators, such as the GnRH pulse generator, vary from the existence of distinct pacemaker cells through to emergent network activity. Single-cell GRIN lens mini-endoscopic imaging is another sophisticated method employed for monitoring intracellular calcium levels in specific neurons, *in vivo*, providing the opportunity to examine the behaviour of multiple individual neurons within a network simultaneously (Resendez *et al.* 2016). In 2022, a pioneering study led by Moore and colleagues technically advanced reproductive neuroendocrine research shedding light on KNDy cell synchronisation at the single-cell level in freely behaving ovariectomised female mice using GRIN lens mini-endoscopy (Moore *et al.* 2022). Synchronous activation of all KNDy cells was observed in the field of view preceding each LH pulse. Large amplitude calcium signals during an endogenous synchronous episode were critical for LH secretion. Interestingly, there were also synchronous episodes observed where not all KNDy cells in the field of view displayed activity; however, in these instances the mean amplitude of calcium signal across cells was lower, and these synchronous episodes were not followed by LH release. The synchronous episodes appeared to be temporally organised with subsets of ‘leader’ and ‘follower’ cells. The subpopulation of ‘leader’ cells displayed a calcium peak first, capable of driving the initiation phase of an episode. The remaining subsets of ‘follower’ cells displayed a calcium peak midway or at the endpoint of the episode. In contrast to leader cells, follower cells are temporally restricted to exhibit calcium activity later within the episode. Albeit analysis of the temporal order within the KNDy network is limited, as it is restricted to the imaging field of the GRIN lens and the development of three-dimensional imaging is required. The *in vivo* implementation of kiss1 neuron-specific electrical recordings or voltage-sensitive indicators would provide precise patterning of KNDy neuronal firing leading up to and during a synchronous episode. The presence of synchronous episodes not followed by pulsatile LH secretion, could be due to the rate of blood sampling, as higher frequency LH pulses may be difficult to capture. Long-term ovariectomy also results in high-frequency GnRH pulses that may desensitise the pituitary to GnRH, leading to reduced LH secretion, like previous observations made in ovariectomised monkeys (Wildt *et al.* 1981) and sheep (McIntosh & McIntosh 1983). Nevertheless, a model was proposed whereby activating leader cells slowly increase in their activity toward a threshold, which depolarises neighbouring KNDy cells, and this is propagated from cell to cell. This proposed ‘leader’ and ‘follower’ model resembles the established calcium dynamics observed in oscillatory beta cells of the pancreas, which underlie the pulsatile secretion of insulin (McIntosh & McIntosh 1983).

However, a recent study by Herbison and colleagues opposed the ‘leader’ and ‘follower’ model, and instead suggested a ‘glutamate two-transition’ model (Han *et al.* 2023). Unlike the study performed by Moore *et al.* this study was performed in intact male mice where synchronous episodes were detected approximately every 180 min, *in vivo*; however, pulsatile LH secretion was not measured. Additionally, low-amplitude ‘miniature synchronous episodes’ were also recorded using GCaMP fibre photometry. Interestingly, there appeared to be a weak correlation for the order of activation of individual KNDy neurons, indicating there may be variable strengths of coupling among microcircuits of KNDy neurons, which contrasts with the study performed by Moore *et al.* where a tighter correlation was reported for the order of activation of KNDy neurons in a synchronous episode (Moore *et al.* 2022). This disparity could be due to the application of different animal models where in the study by Moore *et al.* ovariectomised female mice were used whereas the study by Han *et al.* utilised intact male mice. Han *et al.* have shown that glutamate is the key neurotransmitter enabling synchronous activity within the KNDy network by directly infusing glutamate antagonists in the ARC, which resulted in an inhibition of synchronous episodes (Han *et al.* 2023). However, administration of glutamate antagonists into the ARC may affect neighbouring cells that also utilise glutamate neurotransmission and communicate with KNDy neurons, thus possibly affecting the observations obtained from these recordings. Regardless, these observations agree with previous reports by Qiu *et al.* and Voliotis *et al.*, which established the critical involvement of glutamate signalling in the synchronous activity of the KNDy network (Qiu *et al.* 2016, 2018, Voliotis *et al.* 2021). Han *et al.* proposed that glutamate initiates the first transition stage in the model whereby glutamatergic excitation among KNDy neuronal assemblies leads to the exponential recruitment of neurons in the network to achieve the synchronous episode (Han *et al.* 2023). The efficacy of this initial transition is modulated by a dynorphin-κ-opioid tone within the network as well as any external inputs to the KNDy network, which are also involved in regulating its frequency of activity. Administration of norBNI, a κ-opioid receptor antagonist, into the ARC, appeared to have no effect on the interval of synchronous episodes in gonadectomised mice, whereas norBNI treatment initiated synchronous episodes in intact male mice. Similarly, norBNI administration in the ARC has no effect on LH pulses in ovariectomised rats (Grachev *et al.* 2012) but enhances LH pulse frequency in ovariectomised ewes (Goodman *et al.* 2013). Moreover, in the clinic, administration of naloxone (opioid antagonist) is a robust strategy for increasing LH pulses (Perkins *et al.* 1999, Tenhola *et al.* 2012, Lippincott *et al.* 2019). Dynorphin tone within the network possibly has a limited role in the free-running GnRH pulse generator state (Bhanot & Wilkinson 1984, Melis *et al.* 1984, Whisnant & Goodman 1988, , Mostari *et al.* 2013), but dynorphin-κ-opioid transmission suppresses generation of synchronous episodes in the presence of the gonads. The second transition in the model involves NKB mediated potentiation of existing glutamate-driven excitation to reach maximum activity by increasing the number of KNDy neurons involved in a synchronous episode (Han *et al.* 2023). Lastly, intrinsic mechanisms governing termination of a synchronous episode were proposed. Since dynorphin was not found to play a part in the termination of KNDy neuronal synchronisation, calcium-activated potassium channels are suggested to operate in the KNDy network to limit firing frequency in times of sustained activation; however, more work is required to determine the exact mechanism involved in the termination of KNDy neuronal synchronisation. In addition, intense KNDy network firing would be followed by reduced glutamate and NKB release; thus, this was proposed as another possible contributing factor to the termination of synchronous episodes (Han *et al.* 2023).

The application of calcium imaging *in vivo* permitted intracellular calcium activity of KNDy neurons to be measured in real time. KNDy neurons were shown to exhibit brief synchronised episodes of calcium activity perfectly correlated with LH pulses in intact and gonadectomised mice, reinforcing the role of the KNDy network as the GnRH pulse generator. The inter-synchronisation episode interval was drastically reduced in the absence of gonadal steroids compared to intact mice. Interestingly, a wide range of inter-synchronisation episode intervals were observed in both intact and gonadectomised mice, indicating an unpredictable stochastic pattern of GnRH pulse generator activity. Intriguingly, long-term gonadectomised mice display synchronised episodes with higher levels and multi-episode synchronisations compared to intact as well as short-term term gonadectomised mice. This difference in activity likely involves ESR1 signalling, as selective deletion of ESR1 from KNDy neurons results in GnRH pulse generator activity equivalent to that of gonadectomised mice. Further application of calcium imaging at the single-cell level has shed light on the origin of episodic activity of the GnRH pulse generator, although two different models have been developed based on the observations from these studies. In the first model, activating leader cells slowly increase in their activity toward a threshold, which depolarises neighbouring KNDy cells, and this is propagated from cell to cell. The second model was termed the ‘glutamate two-transition’ model. The first transition is initiated by glutamate whereby glutamatergic excitation among KNDy neuronal assemblies leads to the recruitment of nearby neurons in the network to achieve the synchronous episode. The efficacy of this initial transition is modulated by a dynorphin-κ-opioid tone within the network and likely via external inputs to the KNDy network. The second transition in the model involves NKB mediated potentiation of existing glutamate-driven excitation to reach maximum activity by increasing the number of KNDy neurons involved in a synchronous episode.

## Conclusion

The combination of cutting-edge approaches has been pivotal in our quest to unravel the nature and function of the GnRH pulse generator. Electrophysiological studies have revealed that KNDy neurons display inherent irregular firing patterns influenced by fast inactivating potassium currents and voltage-gated potassium channels. The circuit dynamics regulating the episodic activity of the KNDy neuronal network involve the action of glutamate, NKB, and dynorphin signalling within the network (Fig. 1). Additionally, the excitability of KNDy neurons is regulated by STIM1, contributing to their synchronous firing. The KNDy network is also steroid sensitive, and the presence of oestradiol has been shown to regulate intra-network communication by modifying glutamatergic transmission to KNDy neurons as well as modulating voltage-gated potassium currents in KNDy neurons (Fig. 1). Optogenetic investigations have further clarified the KNDy network's role as the GnRH pulse generator. These studies have contributed to unravelling the mystery behind what initiates GnRH pulse generation revealing that modulation of the basal activity of the KNDy neuronal population induces changes in the oscillatory behaviour of the KNDy network. Moreover, NKB was confirmed to provide positive feedback and dynorphin slow negative feedback to generate robust oscillation of neural activity in KNDy neurons. Further mathematical modelling combined with optogenetic experimentation supports the idea that the KNDy network’s oscillatory behaviour is sensitive to gonadal steroids, particularly influencing glutamate activity and inter-neuronal communication. Calcium imaging techniques have allowed for real-time monitoring of KNDy neuron activity. These neurons exhibit synchronised calcium oscillations at the neonatal stage where signalling via the NMDA and GABA_A_ receptors plays an essential role. The output signal produced from KNDy network activity is the release of kiss1, which activates distal GnRH dendrons via short-distance volume transmission. In addition, GABAergic signalling also contributes to modulating the distal GnRH dendron further controlling GnRH secretion. Calcium imaging of KNDy neurons, *in vivo*, has confirmed the correlation between KNDy neuron activity and LH pulses, with the interval between synchronisations influenced by circulating gonadal steroids. More recently, the application of calcium imaging on the single-cell level has contributed to our understanding of the origin of episodic activity of the GnRH pulse generator. Two models have emerged: the ‘leader’ and ‘follower’ model, and the other, the 'glutamate two-transition' model. These findings collectively enhance our comprehension of the KNDy network's role in the regulation of reproductive hormones. The continued application of these approaches and others in the future will significantly advance our understanding of KNDy neuronal function in normal physiology, while also having major implication for clinical research providing better treatment strategies for reproductive pathophysiology and infertility.

## Declaration of interest

The authors have nothing to disclose.

## Funding

This work did not receive any specific grant from any funding agency in the public, commercial or not-for-profit sector.

## Author contribution statement

DI, Writing – original draft; KOB, Writing – review and editing.
